# Recognition of Mental Illness among Primary Care Providers in Hungary: Strengths and Limitations

**DOI:** 10.1192/j.eurpsy.2025.872

**Published:** 2025-08-26

**Authors:** V. Swisher, D. Ori, R. Wernigg

**Affiliations:** 1 Psychology, The Pennsylvania State University, State College, United States; 2 Psychology, Semmelweis University; 3 Psychology, Heim Pal National Pediatric Institute; 4 National Directorate-General for Hospitals, Budapest, Hungary

## Abstract

**Introduction:**

With 1 in every 8 people living with a mental disorder according to the World Health Organization, the need for appropriate identification and treatment of mental health conditions is paramount. As the majority of people with mental health problems seek help and receive their mental health care from primary care providers (PCPs), PCPs assume an important role in the identification of mental illness.

**Objectives:**

This study examined mental health literacy and predictors of disorder recognition among primary care providers (PCPs) in Hungary.

**Methods:**

Hungarian PCPs (n = 208) completed a survey assessing demographics, mental health stigma, and exposure to mental health. Participants read six vignettes describing obsessive-compulsive disorder (OCD) harm/aggression subtype (OCD-Aggression), OCD order/symmetry subtype (OCD-Order), generalized anxiety disorder (GAD), social anxiety disorder (SAD), panic disorder (PD), and major depressive disorder (MDD) and were asked to identify each condition and provide treatment referrals. Descriptive analyses were used to characterize disorder recognition rates, perceived disorder causes, and treatment referrals. Binary logistic regression analyses were conducted to examine the degree to which demographic characteristics, mental health stigma, and exposure to mental health predict accurate disorder recognition.

**Results:**

Identification rates for each vignette were: OCD-Aggression (27.9%), OCD-Order (75.5%), SAD (34.1%), GAD (76.0%), PD (78.8%), and MDD (91.3%). First-choice treatment referrals were a psychiatrist for OCD-Aggression (63%), OCD-Order (53.8%), and MDD (46.6%), a psychologist/therapist for SAD (58.7%) and GAD (48.6%), and a PCP for PD (39.9%). Anxiolytics (e.g., benzodiazepines) were the most commonly recommended medication for the anxiety disorders. Mislabeling conditions was significantly associated with older age (for GAD, OCD-Aggression, PD and MDD), male gender (for GAD), greater mental health stigma (for OCD-Order), and not having a family member/friend with a mental health condition (for SAD).

**Conclusions:**

Findings highlight strengths (e.g., depression recognition) and limitations (e.g., OCD-Aggression) in knowledge of mental health conditions among PCPs in Hungary. Our findings add to the literature by outlying potential intervention targets (e.g., increasing education on appropriate anxiolytic use) to improve mental health literacy in primary care. Future research should investigate the efficacy of psychoeducation interventions, particularly for OCD and anxiety disorders, in improving the mental health literacy of PCPs in Hungary.

**Disclosure of Interest:**

V. Swisher Grant / Research support from: This work was supported in part by U.S. Student Fulbright Association., D. Ori: None Declared, R. Wernigg: None Declared

